# Supplementary material for: Influence of Device Geometry and Imperfections on the Interpretation of Transverse Magnetic Focusing Experiments
Source: Nanoscale Res Lett. 2022 Mar 5;17:31. doi: 10.1186/s11671-022-03671-x (PMC8898212; doi:10.1186/s11671-022-03671-x)
Supplement: Supplementary file 1 — Additional file 1. Derivation of mean free path formula (Eq. 11). [file 11671_2022_3671_MOESM1_ESM.zip › TMF_Supplementary.pdf]

NANO EXPRESS

Supplementary Information

Yik Kheng Lee[1,2*], Jackson S. Smith[1,2] and Jared H. Cole[1,2]

*Correspondence: yik.kheng.lee@student.rmit.edu.au
[1]Theoretical, Computational, and Quantum Physics, School of Science, RMIT University, Melbourne, Australia
Full list of author information is available at the end of the article

1 Derivation of mean free path formula (Eq. 11)

Here we derive Eq. 11. We begin with an equation for the scattering time in the single subband case (τ_0) given in pg. 168 of [1]:

$$\frac{\hbar}{\tau_0} = n_i \frac{m^*}{2\pi\hbar^2} \int_0^{2\pi} d\varphi \left\langle \left| v^{(i)}(\vec{q}) \right|^2 \right\rangle_{\text{imp}} (1 - \cos\varphi) \tag{1}$$

where n_i is the areal density of scatterers, φ is the scattering angle, and $\left\langle \left| v^{(i)}(\vec{q}) \right|^2 \right\rangle_{\text{imp}}$ is the ensemble average of the squared scattering potential matrix elements.

The areal density of scatterers is defined as:

$$n_i := \frac{N_i}{A} \tag{2}$$

where N_i is the number of scattering centers within the normalization area A.

In our disorder simulations we applied an onsite potential to every discretisation site in the device, which act as the scatterers. Since there is always one scatterer within each unit cell in the discretisation grid, our normalization area is the area of one unit cell, a^2, where a is the grid spacing length. Therefore our areal density of scatterers is one per unit cell area:

$$n_i = \frac{1}{a^2} \tag{3}$$

The ensemble average of the squared scattering potential matrix elements can be related to the ensemble average of the squared scattering matrix elements by [1]:

$$\left\langle \left| v^{(i)}(\vec{q}) \right|^2 \right\rangle_{\text{imp}} = \frac{A^2}{N_i} \left\langle \left| \langle m\mathbf{k}_m | V | n\mathbf{k}_n \rangle \right|^2 \right\rangle_{\text{imp}} \tag{4}$$

where V is the scattering potential. Since our scattering potential is a uniform random distribution within the range $\left[-\frac{U}{2}, \frac{U}{2}\right]$, independent of $\mathbf{k}$, $\langle m\mathbf{k}_m | V | n\mathbf{k}_n \rangle = V$. The probability distribution of V, $f_x(V)$ is given by:

$$\begin{aligned} f_x(V) &= \frac{1}{V_{max} - V_{min}} \\ &= \left[\frac{U}{2} - \left(-\frac{U}{2}\right)\right]^{-1} \\ &= \frac{1}{U} \end{aligned} \tag{5}$$

Applying the law of the unconcious statistician to Eq. 4, we obtain:

$$\begin{aligned}\left\langle \left| v^{(i)}(\vec{q}) \right|^2 \right\rangle_{\text{imp}} &= \frac{A^2}{N_i} \left\langle |V|^2 \right\rangle_{\text{imp}} \\ &= \frac{A}{n_i} \int_{-\frac{U}{2}}^{\frac{U}{2}} V^2 f_x(V) dV \\ &= \frac{a^2}{n_i} \frac{U^2}{12} \end{aligned} \tag{6}$$

We can rewrite the scattering time in terms of the mean free path length l_{mfp} and Fermi velocity v_{F}:

$$\begin{aligned}\tau_0 &= \frac{l_{\text{mfp}}}{v_{\text{F}}} \\ &= l_{\text{mfp}} \sqrt{\frac{m^*}{2E_{\text{F}}}} \end{aligned} \tag{7}$$

Substitute Eqs. 3, 6 and 7 into Eq. 1:

$$\begin{aligned}\frac{\hbar}{\tau_0} &= n_i \frac{m^*}{2\pi\hbar^2} \int_0^{2\pi} d\varphi \left\langle \left| v^{(i)}(\vec{q}) \right|^2 \right\rangle_{\text{imp}} (1 - \cos\varphi) \\ \frac{\hbar}{l_{\text{mfp}}} \sqrt{\frac{2E_{\text{F}}}{m^*}} &= n_i \frac{m^*}{2\pi\hbar^2} \frac{a^2}{n_i} \frac{U^2}{12} 2\pi \\ l_{\text{mfp}} &= \sqrt{\frac{2}{m^*}} \frac{12\hbar^3}{m^* a^2} \frac{\sqrt{E_{\text{F}}}}{U^2} \end{aligned}$$

We want to express the energy terms as fractions of the hopping energy t.

$$\begin{aligned}l_{\text{mfp}} &= \sqrt{\frac{2}{m^*}} \frac{12\hbar^3}{m^* a^2} \frac{\sqrt{E_{\text{F}}/t}}{U^2/t^2} \frac{\sqrt{t}}{t^2} \\ &= 48a \frac{\sqrt{E_{\text{F}}/t}}{U^2/t^2} \end{aligned} \tag{8}$$

Author details
[1]Theoretical, Computational, and Quantum Physics, School of Science, RMIT University, Melbourne, Australia.
[2]ARC Centre of Excellence in Future Low-Energy Electronics Technologies, RMIT University, Melbourne, Australia.

References

1. Ihn T. Semiconductor Nanostructures. Oxford University Press; 2009.
